# Significance of Gut Microbiota and Short-Chain Fatty Acids in Heart Failure

**DOI:** 10.3390/nu14183758

**Published:** 2022-09-11

**Authors:** Peng Zhao, Suhong Zhao, Jinwei Tian, Xinxin Liu

**Affiliations:** 1 Department of Cardiology, The Second Affiliated Hospital of Harbin Medical University, Harbin 150086, China; 2 Key Laboratory of Myocardial Ischemia, Ministry of Education, Harbin 150086, China

**Keywords:** heart failure, gut microbiota, short-chain fatty acids, treatment

## Abstract

Heart failure (HF), as the terminal stage of various heart diseases, seriously threatens an individual’s life, health, and quality of life. Emerging evidence has shown that the gut microbiota comprises an important component of human physiology and metabolic homeostasis, and can directly or indirectly affect the metabolic health of the host through metabolites. Upon in-depth study of intestinal microecology, the “gut-heart axis” appears to provide a novel direction for HF research. Thus, this review primarily focuses on the relationship between the gut microbiota and its major metabolites—i.e., short-chain fatty acids (SCFAs)—and HF. It explores the mechanisms underlying HF and its effective treatment by targeting SCFAs to optimize current HF treatment and thus improve the quality of patients’ lives.

## 1. Introduction

Heart failure (HF) is defined as a clinical syndrome caused by depressed cardiac output or elevated ventricular filling pressure that results in systemic neurohumoral dysfunction and multi-organ involvement [1,2]. The number of HF patients is currently increasing due to an aging population, global population growth, and improvements in survival after diagnosis. Today, there are approximately 64.3 million people with HF worldwide, and its prevalence in developed countries has risen to 1–2% of all adults [3]. As a result of adverse factors such as household air pollution, poor diet, and low education levels in low-and middle-income countries, additional challenges are anticipated in the treatment of HF [4]. In China, for example, the prevalence of HF in adults aged 35 years and older increased by 44% during the past 15 years [5]. In the urban HF patients, the average outpatient cost per capita is $892, with readmissions increasing with age, and the mean annual inpatient cost is as high as $4406 [6]. This increasing burden is therefore taking a great toll on society.

Although the rational use of HF drugs—including angiotensin receptor-neprilysin inhibitors, β-adrenergic receptor antagonists, mineralocorticoid receptor antagonists, and sodium-glucose cotransporter-2 inhibitors—can robustly reduce morbidity and mortality in the majority of patients with HF, [2] the economic burden of the non-elderly living with HF has grown following a surge in obesity and obesity-related diseases and constitutes an alarming and ominous sign [3,7]. Accordingly, it is critical that we actively search for a new and effective therapy to counteract HF.

## 2. Text

### 2.1. Gut Microbiota

The gut is a gatekeeper to human nutrient absorption, and the gut microbiota is increasingly shown to be vital to gut health [8]. The composition of gut microbiota includes bacteria, archaea, fungi, and certain protists and viruses, [9] and these microbes are estimated to collectively comprise up to 100 trillion cells (which is 10 times the number of human somatic cells) and to encode 100 times as many unique gene products relative to the genome in our own bodies. Over 99% of the microbiotal genes are bacterial, indicating that bacteria occupy a dominant position in the gut [10]. Such a vast microbiota contains the body’s densest ecosystem and functions in physiological roles by modulating nutrition, the immune system, and host defenses [8].

Specific compounds in breast milk drive and modulate the establishment of the intestinal microflora in humans from birth [11]. Following bodily growth, both microbial abundance and the microbial genome exhibit a certain degree of intra-individual long-term stability [12]. Thus, the physiological progression of the gut microbiota from its initiation—from meager to enriched and from simple to complex—can be said to comprise a symbiotic relationship between the gut microbiota and humans [11].

### 2.2. Disturbances of the Gut Microbiota

Our current thinking reflects a marked departure from previously held positions in that the gut microbiota is not in direct contact with the intestinal endothelium, but is rather separated by a layer of mucus that possesses lubricating and hydrating properties [13]. The mucus layer plays an important role in protecting the intestine from mechanical, chemical, and biological attacks, and the colonic microbiota normally fails to penetrate the inner mucus layer. However, with gut microbiotal dysbiosis caused by host diet or other factors, the microbiota will inevitably be found to be in direct contact with the intestinal endothelium, leading to certain diseases [14].

Evidence points to an association between disturbances in the gut microbiota and diseases such as cardiovascular disease, diabetes, Alzheimer’s disease, inflammatory bowel disease, obesity, cancer, and the novel coronavirus infection, COVID-19 [8,15,16,17,18,19,20]. It is noteworthy that regardless of whether patients with COVID-19 received medication, their gut microbiota composition changed significantly [21]. It was confirmed that gut microbiotal dysbiosis can impair responses between the vaccine and antibodies, and may therefore suggest an adverse impact on the development and application of the SARS-CoV-2 vaccine [22].

Briefly, the gut microbiota relies on various biomolecules, nutrient signals, and epigenetic mechanisms to communicate with the host, and thereby enacts a favorable impact on host health. In contrast, perturbation of the gut microbiota disrupts the interdependence between the gut microbiota and host, and tilts the physiological balance of health to the other side [23].

### 2.3. Changes in the Gut Microbiota of HF Patients

There exists a correlation between HF and gut microbiota. 16S rRNA-amplicon sequencing has evolved into an important method used to study the composition and distribution of the gut microbial community, and provides more comprehensive knowledge and awareness of microbial diversity and its complex physiological traits [24]. Similar to the situation in healthy individuals, the gut microbiota in HF patients is dominated by Firmicutes and Bacteroidetes, followed by Proteobacteria, [25] and many studies have shown that the composition of the gut microbiota in HF patients is altered considerably, [26] as depicted in Table 1. Significant modifications to the ratio of Firmicutes to Bacteroides (F/B) were observed in the gut microbiota of some HF patients, implicating the gut microbiota as an emerging and novel biomarker in the prediction of HF [27,28,29].

The changes to gut microbiota may also be different for disparate types of HF. A con- trolled clinical trial confirmed that the number of *Rominococcus* spp. in patients showing heart failure with preserved ejection fraction (HFpEF) was markedly reduced [29]. Another study on the relative bacterial abundances of the common core genera in patients with heart failure with reduced ejection fraction (HFrEF) revealed that *Streptococcus* and *Veillonella* were enriched, while SMB53 was reduced [25]. In addition, as HF gradually progressed, gut microbiota also changed accordingly. Investigators demonstrated that gut microbiotal diversity in patients with New York Heart Association (NYHA) Class IV HF was significantly attenuated; that endotoxemia, inflammation, and oxidative stress were augmented; and that gut microbiota with potential anti-inflammatory effects was enriched in patients with Class I and II HF [30]. Similar results were also reported in two other studies, with an increase in *Escherichia* and *Shigella* in the HF transformation from a compensated to a decompensated state. A positive correlation between the abundances of *Escherichia* and *Shigella* and the deleterious metabolites trimethylamine N-oxide (TMAO) and indoxyl-sulfate (IS) implies that the gut microbiota may also contribute to HF [31,32].

Aging, as an independent and inevitable risk element, is the predominant risk factor for cardiovascular disease, and along with other factors, it collectively modulates the development of the disease [33]. Kamo et al. concluded that the gut microbiota of HF patients changed with age, with the proportion of Bacteroidetes diminishing and the quantity of Proteobacteria increasing in elderly HF patients [34]. Subsequent authors also determined that the percentage of Actinomycetes in the gut of aged mice fed an obesogenic diet (OBD) was further enhanced relative to that of younger mice [35].

### 2.4. Correlation between Gut Microbiota and HF

The integrity of intestinal structure and function is inextricably linked with disease, and serum lipopolysaccharide (LPS) and zonulin are considered to be two important indicators of intestinal barrier function and intestinal permeability. One study depicted the levels of these molecules as significantly increased in the blood circulation of hypertensive HF mice, and congruent with this observation, the pathology also revealed that the loss of colonic mucosal integrity was accompanied by inflammatory cell infiltration [27]. LPS reduces ZO-1 tight junctions (TJs) in a Toll-like receptor 4 (TLR4)-dependent manner and induces an apparent deformation of intestinal epithelial TJs, causing destruction of the integrity of the intestinal barrier; this “leaky gut” then engenders an aberrant interaction between luminal contents and the intestinal mucosa [36,37].

In HF patients who manifest a microbiotal disorder of the mucosal epithelium, LPS (the primary inflammatory trigger) can then transit through damaged intestinal mucosa into the systemic circulation. While LPS directly acts on cardiac myocytes and macrophages to release various proinflammatory cytokines through the stimulation of TLR4, [38] this activity is exacerbated in HF patients [39]. In addition, NLRP3-inflammasome activation is indispensable to myocardial injury, and interleukin-β (IL-β) and IL-18 are the downstream factors of the NLRP3 inflammasome [40]. Changes in the levels of the corresponding metabolites TMAO and short-chain fatty acids (SCFAs) caused by the reduction in intestinal probiotics or dysbacteriosis can activate the NLRP3 inflammasome and thereby affect the secretion of inflammatory cytokines [41,42]. In summary, HF-induced tissue hypoperfusion, ischemia, and edema within the intestines, intestinal epithelial dysfunction, and intestinal barrier weakness produce bacterial translocation and induce systemic low-grade inflammation.

**Figure 1:**
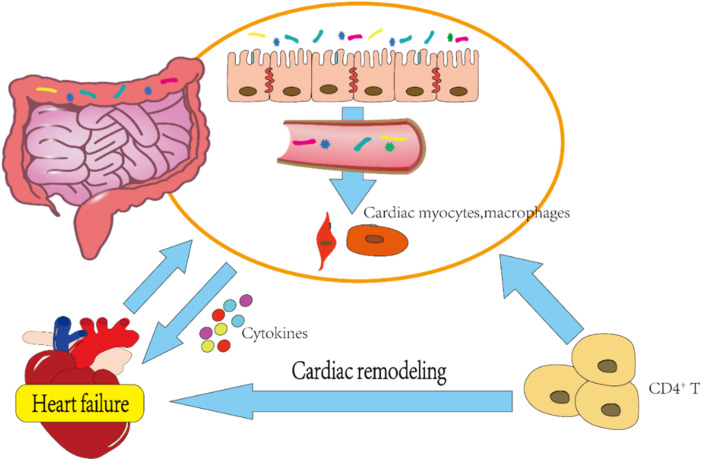
Correlation between gut microbiota and HF. The gut microbiota of patients with heart failure is disturbed, leading to the destruction of intestinal conjunction and the impairment of intestinal mucosal integrity. The gut microbiota entering the blood binds to the Toll-like receptor 4 (TLR4) on the surface of cardiac myocytes and macrophages, promotes the secretion of proinflammatory cytokines, and further aggravates heart failure. In addition, the gut microbiota aggravates cardiac remodeling by influencing immune cells, such as CD4^+^ T cells, and affects the progression of heart failure.

In addition, the gut microbiota interacts with the host immune system in a cyclic manner, [8] with one study showing that the adhesion of segmented filamentous bacteria (SFB) to intestinal epithelial cells (ECs) promotes TH17 cell development [43]. The intestinal commensal colonization of *Bacteroides fragilis* can activate the TLR pathway to induce regulatory T cells and ultimately establish host–microbe symbiosis [44]. CD4^+^ T cell activation is known to be the major cause of cardiac remodeling and fibrosis, and cardiac pressure overload-induced HF leads to specific T-cell activation; whereas the absence of gut microbiota significantly attenuates CD4^+^ T cell number [45]. Figure 1 illustrates the gut microbiota, inflammation, and immunity as intertwined processes. Chronic persistent inflammation and immune response eventually generate related cardiac-function suppression, forming a positive-feedback pathway and aggravating HF.

### 2.5. Correlations between SCFAs and HF

SCFAs are saturated fatty acids with six or fewer carbon molecules that are principally produced by the gut microbiota from resistant starch and dietary fiber (such as cellulose, lignin, and pectin) by fermentation and include acetic acid, acrylic acid, butyric acid, valeric acid, and caproic acid [46]. Broadly speaking, SCFA formation in the colon is largely determined by the type and number of microorganisms, substrate availability, and intestinal transit [47]. SCFAs are among the most abundant microbial metabolites in the intestinal lumen and comprise important energy substances in the human body. SCFAs are absorbed and metabolized by colonic ECs and provide approximately 10% of the daily energy requirement of adults, with acetate as the primary energy source [48].

**Figure 2:**
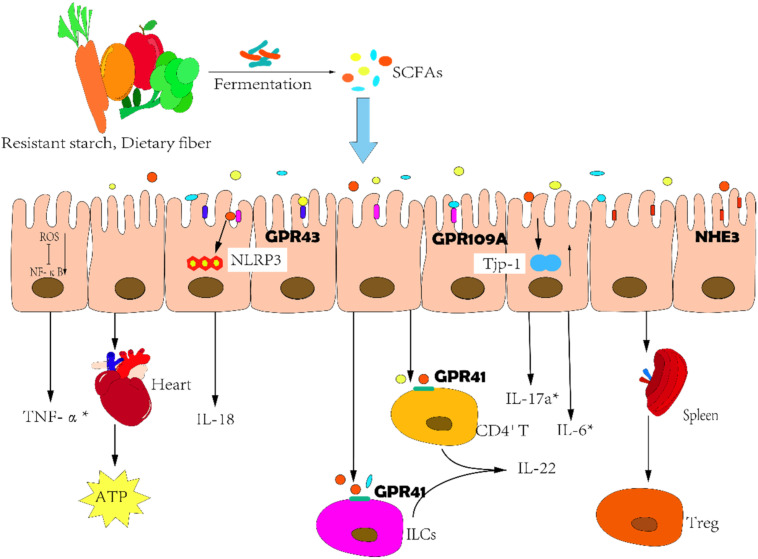
Correlation between SCFAs and heart failure. Short-chain fatty acids (SCFAs) inhibit intestinal and systemic inflammation by binding to G-protein receptor 43 (GPR43), G-protein receptor 109A (GPR109A) and G-protein receptor 41 (GPR41) on the surface of a variety of cells. The secretion of pro-inflammatory factors decreased while anti-inflammatory factors such as IL-22 increased. Moreover, the nuclear transcription factor kappa-B (NF-κB) signaling pathway and immune cells are also involved in the anti-inflammatory process of SCFAs. Apart from this, SCFAs can also enhance myocardial energy metabolism, maintain metabolic homeostasis, and play a cardioprotective role. * denotes decrease; SCFAs, short-chain fatty acids; GPR43, G-protein receptor 43; GPR109A, G-protein receptor 109A; GPR41, G-protein receptor 41; NHE3, sodium–hydrogen exchanger-3; ROS, reactive oxygen species; NF-κB, nuclear transcription factor kappa-B; NRP3, NLRP3 inflammasome; Tjp-1, tight-junction protein-1; TNF-α, tumor necrosis factor-α; IL-18, interleukin-18; IL-17a, interleukin-17a; IL-6, interleukin-6; IL-22, interleukin-22; ILCs, innate lymphoid cells; Treg, regulatory T cell; ATP, adenosine triphosphate.

There exists a vicious cycle that encompasses SCFAs and HF. The sodium–hydrogen exchanger-3 (NHE3) is considered to be the major regulator of sodium and fluid homeostasis in the gut, and the intestinal congestion caused by right ventricular (RV) failure promotes the expression of NHE-3. This action reduces the local pH of the gut and alters the intestinal microenvironment, resulting in the disturbance of the gut microbiota [49]. In HF patients, the abundance of Lachnospiraceae family members diminishes, including several species that produce butyrate; and the abundance of *Muribaculaceae* correlating positively with propionate also decreases [27]. However, SCFAs can mediate interactions with the host SCFA receptors to influence gut health, the immune system, energy metabolism, and cardiovascular function (Figure 2).

**Figure 3:**
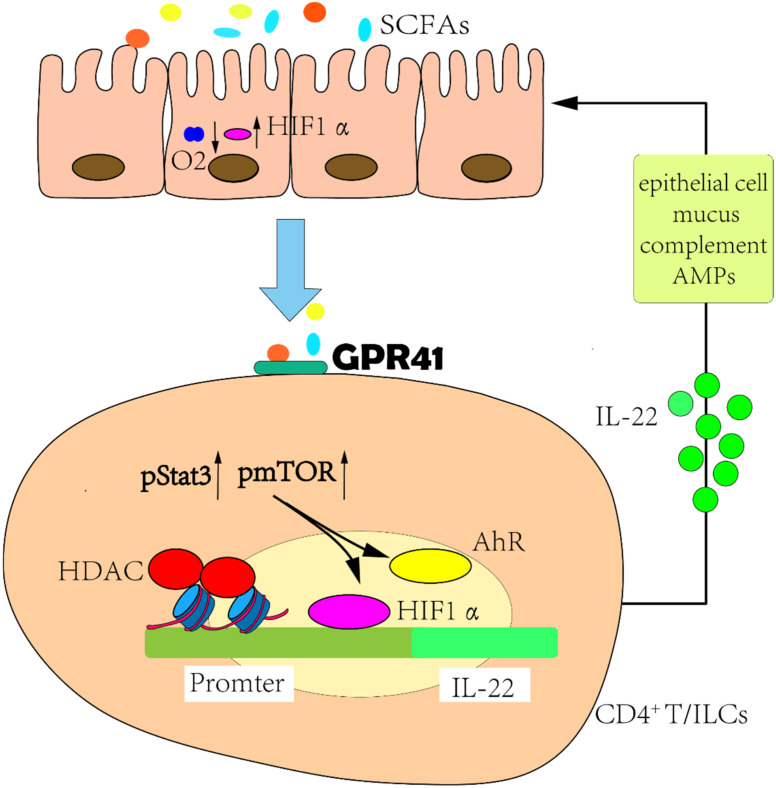
SCFAs promote the production of IL-22. Short-chain fatty acids (SCFAs) promote the expression of hypoxia-inducible factor 1α (HIF1α) and binding to the interleukin-22 (IL-22) by binding to G-protein receptor 41 (GPR41) on the surface of innate lymphoid cells (ILCs) and CD4^+^ T cells and inhibiting histone deacetylase. Finally, the elevated secretion of IL-22 further improves intestinal function. ↑ denotes increase; ↓ denotes decrease; SCFAs, short-chain fatty acids; GPR41, G-protein receptor 41; IL-22, interleukin-22; AhR, aryl hydrocarbon receptor; HIF1α, hypoxia-inducible factor 1α; HDAC, histone deacetylase; ILCs, innate lymphoid cells; AMPs, antimicrobial proteins.

The development of HF is accompanied by an imbalance between inflammatory and anti-inflammatory cytokines [50]. SCFAs regulate the secretion of cytokines via several white blood cell types [51] and also mediate the function of a variety of immune cells—including mast cells that participate in immune responses and local inflammatory reactions [52]. In fact, butyrate binds to G-protein receptor 41 (GPR41) to augment the phosphorylation levels of mTOR and Stat3 and induces the expression of aryl hydrocarbon receptors (AhR) and the transcription factor hypoxia-inducible factor 1α (HIF1α). Butyrate also inhibits histone deacetylase (HDAC), resulting in the acetylation of HREs on the cytokine interleukin-22 (IL-22) promoter to promote the binding of HIF1α, and ultimately enhances IL-22 production by human CD4^+^ T cells and innate lymphoid cells (ILCs) [53]. Researchers have confirmed that IL-22 promotes epithelial barrier function by regulating epithelial cell growth and permeability, mucus production, and the synthesis of antimicrobial proteins (AMPs) and complement—thus preventing intestinal inflammation [54]. Furthermore, HIF1α is also a protective intestinal barrier factor, and SCFAs can enhance O_2_ consumption in intestinal ECs and stabilize the transcriptional activity of HIF1α. In antibiotic-treated or germ-free mice, the low expression levels of HIF1α are restored by butyrate supplementation [55] (Figure 3). Apart from this, butyrate also reduces reactive oxygen species (ROS)-mediated nuclear transcription factor kappa-B (NF-κB) activation and thereby modulates the expression of ICAM-1, COX-2, and TNF-α in intestinal ECs challenged with LPS [56]. In another related study, SCFAs were reported to exert a cardio-protective effect by mediating cognate G-protein receptor 43 (GPR43) and 109A (GPR109A) so as to alter DNA methylation and regulate catecholamine precursor L-3,4-dihydroxyphenylalanine (L-DOPA) levels and regulatory T cell (Treg) abundance of spleen [57]. Within the gut, acetate significantly increased tight-junction protein-1 (Tjp-1) mRNA levels and inhibited the expression of the pro-inflammatory cytokines interleukin-17a (IL-17a) and IL-6. Propionate depends upon Treg to an extent to reduce systemic inflammation, myocardial hypertrophy, fibrosis, and vascular dysfunction [57,58].

Since energy metabolism is critical to the development of HF and the heart in HF is energy-starved, SCFAs are hypothesized to exert a significant influence on host energy metabolism [59]. For example, butyrate perfusion significantly improved mitochondrial adenosine triphosphate (ATP) synthetic efficiency and contractile function in rats fed a high-fat, high-sucrose (HFHS) diet, thereby correcting the cardiac energy starvation and decreased systolic function caused by the HFHS [60]. This corrective effect may have been due to the elevated levels of acyl coenzyme A synthetase medium-chain family member 3 (ACSM3) enzyme, a mitochondrial enzyme that is normally under-expressed in the heart. ACSM3 enhances butyrate oxidation, effectively supporting energy production in rat HF and in human HF [61]. Propionate stimulates sympathetic neurons through the GPR41 receptor and thus directly enhances SNS outflow, controlling energy consumption and maintaining metabolic homeostasis [62]. These results portend the adoption of SCFAs as a potential treatment for HF, and, intriguingly, SCFAs as substrates do not affect apoptosis or IL-6 production [63].

A recent observational study revealed a significant negative association between butyrate concentration and exhaled hydrogen concentration after a breath test that non-invasively assessed small intestinal bacterial overgrowth (SIBO) in patients with acute heart failure (AHF) [64]. Unfortunately, SIBO can increase the risk of HF rehospitalization in patients with HFrEF and the risk of cardiovascular mortality in patients with HFpEF [65]. Importantly, butyrate as an independent factor significantly reduced bacterial overgrowth in the small intestine [64].

HF is the terminal stage of all heart diseases, and any adverse factor that affects heart disease can aggravate HF. Although SCFAs exert overall protective effects on cardiovascular disease, the direct effect of SCFAs on HF still requires further investigation. Francine et al. exploited the deoxycorticosterone acetate (DOCA) model and demonstrated that a diet high in both fiber and acetate reduced the heart-to-body weight ratio and that it normalized cardiac fibrosis and hypertrophy and improved the overall cardiac function in rats [66]. In addition, SCFAs assist in reducing blood pressure and the accompanying cardiac hypertrophy and myocardial fibrosis [57]. Pulmonary hypertension (PH) is a common disease that ultimately leads to right HF, and research has revealed that butyrate supplementation prevents right ventricular hypertrophy (RVH) and hypoxia-induced increased pulmonary vascular remodeling and permeability, delaying the progression of PH and preventing its evolution to HF [67].

### 2.6. SCFAs: A Potential Therapeutic Target

SCFAs are abundant microbial metabolites in the gut, and they have great potential in the treatment of HF. However, achieving the stable production of SCFAs in the intestine and intervening in HF progression are the current foci of attention.

### 2.7. Increased Dietary Fiber Intake

#### The Mediterranean Diet

Dietary therapy constitutes one of the simplest and most convenient and effective treatment modalities used to resist the onset of cardiovascular disease. The Mediterranean diet is one such healthy eating regimen, and it is distinct from the typical Western-type diet characterized by high-calorie, high-fat processed foods [68]. The Mediterranean diet is depicted by an abundance of plant fiber that entails the daily consumption of fruits, vegetables, and grains; a moderate weekly consumption of legumes, nuts, dairy products, fish, and poultry; low-to-moderate amounts of red meat and wine consumed principally with meals; and monounsaturated fatty acids that are derived primarily from olive oil [69]. Fiber intake is closely linked to gut health, and investigators have shown that higher fiber is associated with a higher abundance of genera from the highly polyphyletic class Clostridia, a subset of which can ferment fiber to produce SCFAs in the colon [70].

Although the correlation between the Mediterranean diet and HF is unclear, most studies still support the claim that the Mediterranean diet reduces the incidence or mortality associated with HF (see Table 2 for details) [71,72,73]. Antonino et al. found that adherence to a Mediterranean diet certainly prevents HF but that it also modulates the severity of HF when it does occur, and this may be associated with the underlying pathogenesis of HF [74]. The cardiometabolic benefits of the Mediterranean diet to patients with type 2 diabetes are mostly anti-inflammatory and antioxidative, [75] which may inferentially delay the development of HF patients from the clinical compensatory phase to the decompensated stage. A study by Chrysohoou et al. in 372 HF patients appears to validate this conclusion, as ventricular systolic and diastolic functions were improved in HF patients who adhered to a Mediterranean diet [76]. Another study revealed that a Mediterranean diet improved central hemodynamics, RV function, and arterial stiffness [77].

Dietary interventions, however, are not limited to Mediterranean dietary patterns, as other plant-based, high-fiber diets can be used as environmentally sustainable dietary options that accentuate cardiovascular health and benefit the human body [78].

### 2.8. Adjustments to Gut Microbiota

#### Probiotics and Prebiotics

Intestinal probiotics include *Lactobacillus*, *Bifidobacterium*, *Bacteroides thetaiotaomicron*, *Akkermansia muciniphila*, and a small number of *Escherichia coli* strains. Probiotics can produce cytokines or inhibit apoptosis through specific binding pattern-recognition receptors, the regulation of signaling pathways, and via probiotic metabolites, thereby reducing inflammation and enhancing intestinal epithelial function [79]. Prebiotics are substrates that are selectively utilized by host microorganisms and that confer a health benefit and include indigestible oligosaccharides, fructose, and galactose, which are more likely to be metabolized by *Bifidobacterium* [80].

Pretreatment with a prebiotic complex reversed the intestinal microbial imbalance in rats with HF, and the content of SCFA-producing bacteria such as *Bifidobacterium* and *Propionibacterium* significantly increased, while the LPS content in the circulation was decreased commensurately [81]. Probiotics reduce the production of ROS in cells, thereby inhibiting oxidative stress and heart damage; [82] and oxidized low-density lipoprotein (oxLDL) in serum was significantly lowered in patients with chronic HF who consumed probiotic yogurt for 10 weeks [83]. In a small study, Costanza et al. showed improved left ventricular ejection fraction (LVEF) and decreased left atrial diameter in patients with chronic systolic HF who received the short-term probiotic *Saccharomyces boulardii*, [84] although a recent study revealed no significant effect on LVEF in HF patients treated for three months with *S. boulardii* [85].

### 2.9. Antibiotics

Antibiotics are modulators of the symbiotic relationships between the host and gut microbiota and comprise the most commonly used strategies to modulate gut microbiota in clinical practice, while short-term antibiotic treatment may modulate the gut microbiota to a long-term alternative dysbiotic state and thus allow disease progression and aggravation [86]. Researchers reported that the abundance and diversity of gut microbiota diminished after antibiotic treatment in HF mice, resulting in the expression of biomarker genes involved in cardiac remodeling—including atrial natriuretic factor (ANF), brain natriuretic factor (BNP), and the regulator of calcineurin 1 (Rcan1) [87]. However, some specific antibiotics such as rifaximin (in addition to their bactericidal and antibacterial activity) also promote an elevation in *Bifidobacterium* and lactobacilli in the gut [88]. After vancomycin treatment for seven days, the abundances of butyrate-producing bacteria such as *Coprococcus eutactus* and *Faecalibacterium prausnitzii* were significantly lowered in the gut of metabolically impaired men; but this did not alter gut permeability, bacterial translocation, or the levels of secreted IL-6, IL-8, or TNF-α [89]. Although the use of antibiotics in the treatment of HF is debatable, their potential benefits in the clinical treatment of HF are worthy of further investigation.

### 2.10. Fecal Transplantation

Fecal microbiota transplantation (FMT)—the transfer of feces from a “healthy” donor to a recipient believed to possess altered colonic microbiota that causes disease—is currently the most effective intervention for gut microbiotal disturbances and is also the accepted treatment for recurrent *Clostridium difficile* infection (CDI) [90,91]. Apart from this, FMT has demonstrated potential therapeutic value in other diseases, including ulcerative colitis and autism [92,93]. Microbiological investigations showed that the infusion of feces from healthy donors into the cecum and colon of recipients gradually changed the microbiotal composition of the recipient toward that of the healthy donors and that the newly created microbiotal composition remained stable for approximately 24 weeks [94]. Furthermore, FMT showed a superior therapeutic effect relative to probiotics in mice with dysbiosis and treated with antibiotics, with rapid and nearly complete restoration of the mucosal microbiome and gut transcriptome within days [95]. Although FMT exerts a robust impact on the gut microbiota, its application to HF remains in its experimental stages and requires additional investigation due to the complex procedures and risks inherent to FMT [90].

## 3. Conclusions and Future Perspectives

Accumulating evidence indicates that the gut microbiota is associated with the development of cardiovascular disease. The destruction of the intestinal barrier function in patients with HF leads to bacterial translocation and endotoxin release into the blood, triggering a series of inflammatory and immune responses and aggravating HF. With the increased sophistication of HF treatment options, effectively reducing the morbidity and mortality of HF remains a global focus. In a normal diet, various nutrients are catabolized to distinct metabolites such as TMAO, SCFAs, IS, and aromatic amino acids under the action of gut microbiota; and of these, TMAO is the most widely analyzed with respect to cardiovascular diseases [96,97,98]. However, a role for SCFAs in cardiovascular disease cannot be ignored, and SCFAs actually improve the progression of HF by regulating inflammatory and immune responses and myocardial energy metabolism. Although we expect to attain a deeper understanding of the mechanism (s) underlying SCFA actions in HF, it is currently important that we know that SCFA-centered adjuvant therapy for HF is an effective and aggressive, non-drug-related therapeutic strategy that will reduce the morbidity of HF patients.

## Figures and Tables

**Table 1:** Changes in the gut microbiota of HF patients.

Group						
HF Type	Control	Species	Method	Summary of Results	F/B	Reference
HFrEF (N = 28)	N = 19	Human	16s rRNA	*Streptococcus*, *Veillonella* ↑ SMB53 ↓	_	[25]
CHF (N = 35)	N = 15	Dog	16s rRNA	The abundance of Proteobacteria in patients with CHF increased, primarily Enterobacteriaceae and *Escherichia coli*	_	[26]
HHF (N = 8)	N = 8	Rat	16s rRNA	Ruminococcaceae ↑ Muribaculaceae, Lachnospiraceae, Lactobacillaceae ↓	↑	[27]
HFrEF (N = 84)	N = 266	Human	16s rRNA	Fiber intake influenced gut microbiotal composition and intra-individual diversity	↓	[28]
HFpEF (N = 26)	N = 67	Human	16s rRNA	The gut microbiome of patients with HFpEF showed a depletion of bacteria, particularly *Ruminococcus*	↓	[29]

The HF group is compared with the control group. ↑ denotes increase; ↓ denotes decrease; _ denotes unknown; HF, heart failure; HFrEF, heart failure with reduced ejection fraction; HHF, hypertensive heart failure; HFpEF, heart failure with preserved ejection fraction; CHF, congestive heart failure; F/B, Firmicutes/Bacteroides.

**Table 2:** Adherence to a Mediterranean-style diet reduces HF risk.

Study Population		Country	Research Method	Effects of Mediterranean-Style Diet on HF Events	Reference
Male	Female				
N = 9316	N = 27,645	The Netherlands	Observational study	Adherence to a Mediterranean-style diet reduced HF risk, particularly in men	[71]
N = 33,966	N = 30,713	Sweden	Prospective study	Healthy lifestyle, including adherence to a Mediterranean diet, was correlated with reduced risk for developing HF in both men and women	[72]
N = 37,308	_	Sweden	Prospective study	High adherence to a Mediterranean diet was associated with a 31% reduction in the risk of developing HF	[73]

## Data Availability

Not applicable.
